# Manganese (Mn) Oxidation Increases Intracellular Mn in *Pseudomonas putida* GB-1

**DOI:** 10.1371/journal.pone.0077835

**Published:** 2013-10-17

**Authors:** Andy Banh, Valarie Chavez, Julia Doi, Allison Nguyen, Sophia Hernandez, Vu Ha, Peter Jimenez, Fernanda Espinoza, Hope A. Johnson

**Affiliations:** Center for Applied Biotechnology Studies, Department of Biological Science, California State University Fullerton, Fullerton, California, United States of America; Loyola University Medical Center, United States of America

## Abstract

Bacterial manganese (Mn) oxidation plays an important role in the global biogeochemical cycling of Mn and other compounds, and the diversity and prevalence of Mn oxidizers have been well established. Despite many hypotheses of why these bacteria may oxidize Mn, the physiological reasons remain elusive. Intracellular Mn levels were determined for *Pseudomonas putida* GB-1 grown in the presence or absence of Mn by inductively coupled plasma mass spectrometry (ICP-MS). Mn oxidizing wild type *P. putida* GB-1 had higher intracellular Mn than non Mn oxidizing mutants grown under the same conditions. *P. putida* GB-1 had a 5 fold increase in intracellular Mn compared to the non Mn oxidizing mutant *P. putida* GB-1-007 and a 59 fold increase in intracellular Mn compared to *P. putida* GB-1 ∆2665 ∆2447. The intracellular Mn is primarily associated with the less than 3 kDa fraction, suggesting it is not bound to protein. Protein oxidation levels in Mn oxidizing and non oxidizing cultures were relatively similar, yet Mn oxidation did increase survival of *P. putida* GB-1 when oxidatively stressed. This study is the first to link Mn oxidation to Mn homeostasis and oxidative stress protection.

## Introduction

Bacteria and fungi can catalyze manganese (Mn) oxidation and thus play an important role in the biogeochemical cycling of Mn and other elements. Mn oxidizing bacteria are found in marine, freshwater, and terrestrial environments [1]. These widespread bacteria are found in divergent phylogenetic lineages from Proteobacteria to Firmicutes to the more recently noted Bacteroidetes [2–4]. Biogenic Mn oxidation can occur at much greater rates than homogeneous abiotic oxidation [5] and forms some of the most oxidative compounds in nature, Mn (III, IV) oxides [6]. These oxides can oxidize recalcitrant carbon compounds and reduced metals and sulfide, thereby affecting the fate of many compounds in nature [2,7,8]. Despite the prevalence of Mn oxidizing microbes and their important role in biogeochemical cycling, the physiological function of bacterial Mn(II) oxidation remains enigmatic [9].

*Pseudomonas putida* GB-1 and MnB1, freshwater *Gammaproteobacteria*, are model Mn(II) oxidizing bacteria that have been used for biochemical and molecular investigations into biogenic Mn oxidation. Mutational studies have revealed several genes in *P. putida* that can affect Mn oxidation including genes involved in flagellar synthesis [10], protein transport [11,12], two component response regulation [13], cytochrome *c* biogenesis [14,15], and carbon metabolism [15]. In addition, the oxidizing enzymes, multicopper oxidases, have been identified [16] and partially characterized [17,18]. Yet, the physiological role of Mn oxidation has not been determined. 

Many hypotheses regarding the potential physiological function or benefit of Mn(II) oxidation have been put forth, but strong evidence to support these hypotheses remains lacking [1,2,19]. By acting as an electron acceptor or oxidizing carbon compounds to bioavailable forms, Mn oxides may help the survival of bacteria by playing a role in energy production [20]. In addition, the oxidation of Mn(II) with molecular oxygen to form Mn oxides is an exergonic process and energy from this reaction could potentially be harvested [21]. Mn oxides form extracellularly as a result of Mn oxidation [19] with cells of *Pseudomonas putida* GB-1 becoming encrusted with Mn oxide minerals [17]. In these cases, Mn oxides could act as a protective shield against external reactive oxygen species (ROS), UV light, and predation from protists and viruses [1,22,23]. Mn oxides may also form as a detoxification mechanism to reduce the toxicity of Mn(II) or other toxic metals [1]. The potential benefits and physiological roles could be diverse and may vary between different phylogenetic lineages.

Intracellular Mn has been shown to play an important role in protection against oxidative stress. Archibald and Fridovich [24] initially showed that *Lactobacillus plantarum*, which lacks a superoxide dismutase, could survive aerobic conditions as a result of high levels of intracellular Mn. More recently, Daly et al [25] have shown that *Deinococcus radiodurans* survives high levels of ionizing radiation and oxidative stress as a result of high intracellular Mn and low intracellular iron (Fe). Maintaining Mn homeostasis is an important part of dealing with the dangers of oxidative stress in many different strains of bacteria, and Mn uptake and efflux transporters have been shown to be important in controlling intracellular Mn levels and providing resistance to oxidative stress [26–33]. 

Here we show that Mn oxidation can increase intracellular Mn and can protect against oxidative stress. We propose that Mn oxidation plays a role in Mn homeostasis.

## Methods

### Bacterial strains, media, and growth conditions

*P. putida* GB-1, *P. putida* ∆2665 ∆2447 [16], and *P. putida* GB-1-007 [10,34], the wild type and non Mn-oxidizing mutants, respectively, were grown in Lept media (0.5 g/L yeast extract, 0.5 g/L casamino acids, 5 mM glucose, 0.48 mM calcium chloride, 0.83 mM magnesium sulfate, 3.7 µM iron (II) chloride, 10 mM HEPES pH 7.5, 0.04 µM copper sulfate, 0.15 µM zinc sulfate, 0.08 µM cobalt chloride, 0.06 µM sodium molybdate) [35] with or without 200 µM MnCl_2_. 100 ml cultures were grown at room temperature with shaking (250 rpm) to stationary phase, approximately 24 hours, when maximum Mn oxide precipitation occurred. 

### Preparation of cells for metal analysis

Cells were harvested by centrifugation (11,000g, 20 min, 4° C) using Corning^®^ polypropylene centrifuge tubes and plasticware prewashed in 1 mM EDTA followed by 10% nitric acid. Harvested cells were resuspended in 20 mM ascorbic acid (pH 8) in PBS buffer to reduce manganese oxides with a minimum contact time of 15 minutes. Mn oxides were no longer detected as measured with the Mn oxidation assay (below). Cells were then washed two times in PBS buffer followed by a final wash in 1 mM EDTA in PBS. For ICP-MS analysis, the cell pellet was digested in 1 ml Optima grade nitric acid for 1 hr at 84 °C and then diluted with 34 ml MilliQ water.

### Preparation of cell-free extract and protein separation for metal analysis

Cell pellets were prepared as above without digestion. The cells were lysed by freeze thaw and lysozyme as previously described [36] with minor modifications. Briefly, the washed cell pellets were subjected to 3 cycles of freeze thaw followed by resuspension in 10 mM TRIS pH 7.5, 50 mM sucrose containing 8400 U/ml of Ready Lyse (Epicentre^®^) and incubated for 15 minutes at room temperature. An equal volume of 10 mM TRIS pH 7.5, 50 mM NaCl, 10 mM MgCl_2_ with 12.5 U/ml of Benzonase was then added and incubated another 15 minutes at room temperature. Cells were centrifuged at 4 ° C, 17,000g for 15 minutes and the soluble fraction was defined as the cell-free extract. Cell-free extract was separated into a greater than 3 kDa fraction and a less than 3 kDa fraction using Amicon micro ultrafiltration with a 3 kDa filter according to the manufacturer’s specifications. Ultrafiltration tubes were rinsed once with 1 mM EDTA followed by three rinses with PBS prior to use.

### Survivability assays

Cells were grown in MSTP media modified from [37] containing 20 mM (NH_4_)_2_SO_4_, 0.25 mM MgSO_4_, 0.4 mM CaCl_2_, 0.15 mM KH_2_PO_4_, 0.25 mM Na_2_HPO_4_, 10 mM HEPES, 9 mM sodium pyruvate, 0.01 mM FeCl_3_, 0.02 mM EDTA, and 1 ml/L Trace elements [35]. The cultures were prepared by inoculating an overnight culture with 250 µL of a frozen culture grown in MSTP media. The culture was grown for 24 hours and then 1.25 ml was inoculated into 25 ml of fresh MSTP media with 0 or 0.1 mM MnCl_2_. Cultures were grown for approximately 24 hours until stationary phase when Mn(II) had been converted into Mn oxides. 2 ml of cells were then treated with 16.1 mM hydrogen peroxide for 30 minutes to induce oxidative stress and killing after which 286 U/ml catalase was added to remove remaining hydrogen peroxide. Control cultures were treated with water instead of hydrogen peroxide. Most oxides were reduced upon exposure to hydrogen peroxide, but 0.1 mM ascorbic acid was also added to reduce any remaining Mn oxides that may increase cell clumping. The cells were then serially diluted in MSTP without pyruvate and plated onto MSTP solid media in triplicate. Relative survival was determined by dividing the percent survival of cells grown in 0.1 mM MnCl_2_ (Mn oxidizing) by the percent survival of cells grown without additional Mn added (non oxidizing). Percent survival was calculated as colony forming units (CFUs) of hydrogen peroxide treated cells divided by CFUs of water treated cells. CFUs were averages of triplicates. The one-tailed Student’s T-test was used for statistical analysis.

### Oxidative stress for protein carbonylation assay

Following growth to stationary phase, a 100 ml culture of *P. putida* GB-1 grown on Lept media was stressed with 0.6 mM H_2_O_2_ or 5 mM paraquat for 30 minutes at room temperature. A lower concentration of hydrogen peroxide was used in protein carbonylation assays as opposed to survival assays to decrease death by oxidative stress. After 30 minutes, any remaining Mn oxides in the cultures were reduced with 1 mM ascorbic acid and cells were harvested by centrifugation at 11,000g, 4° C, 10 minutes. The pellets were resuspended in 3 ml 50 mM HEPES pH 7.75. Cells were disrupted by 4 passages through a French press at 18,000 psi and clarified by centrifugation for 20 minutes at 20,000g, 4° C. The extent of protein carbonylation was determined with the OxyBlot (Millipore) kit according to the manufacturer’s specifications. 

### Protein quantification

Protein was determined using the commercial Coomassie Plus Assay reagent (Pierce) with bovine serum albumin as the standard.

### Mn oxidation assay

Oxidized Mn was measured as previously described using leucoberbelin blue with potassium permanganate as the standard [38].

### Inductively coupled plasma mass spectrometry (ICP-MS)

All samples for ICP-MS analysis were acidified with Optima grade nitric acid and analyzed at IIRMES, California State University Long Beach. Triplicate blank samples were prepared in the same manner as all samples for analysis and the average concentration of the various metals in the blanks was subtracted from all samples.

### Chemicals and reagents

All chemicals were reagent grade and purchased from Fisher or Sigma-Aldrich. Milli-Q water was used for the preparation of all solutions.

## Results and Discussion

### Intracellular Mn increases under Mn oxidizing conditions

To determine whether Mn(II) oxidation can play a role in Mn homeostasis, intracellular metal concentrations were determined for *P. putida* GB-1 and two non Mn oxidizing mutants of *P. putida* GB-1 (GB-1-007 and ∆2665 ∆2447). *P. putida* GB-1-007 was previously thought to have a mutation in the multicopper oxidase *cumA* gene which prevented Mn oxidation [34], but has since been found to have an additional mutation in a two component regulatory system sensor kinase which causes the non-oxidizing phenotype [13]. This two component system may regulate more genes than those involved in Mn oxidation as its regulon is currently unknown. *P. putida* ∆2665 ∆2447 is a double mutant with mutations in the *mnxG* and *mcoA* multicopper oxidase genes which are directly involved in catalyzing Mn oxidation [16]. All strains were grown on rich media commonly used for studying Mn oxidizers with and without added Mn (200 µM) for oxidizing and non-oxidizing conditions, respectively. The complex Lept media in the absence of additional Mn contained approximately 1 µM Mn as measured by ICP-MS. Cells were harvested during stationary phase when Mn oxides were most abundant. Mn, Fe, cobalt, copper, nickel, and zinc were determined by ICP-MS. Cobalt concentrations were below or just at the detection level (~0.01 nmoles/mg) in all strains and conditions.

*P. putida* GB-1 showed a significant and greater than 1000 fold increase in intracellular Mn when grown in the presence of Mn (Table 1). There was also an increase in the intracellular concentrations of Fe, copper, nickel and zinc. When grown in the absence of added Mn, P*. putida* GB-1 had similar levels of Mn as *P. putida* KT2440 [25], but Fe levels were much higher (32.7 nmol/mg protein vs 6.8 nmol/mg protein). Although the non-oxidizing mutants also showed an increase in intracellular Mn when grown in media for Mn oxidation, the intracellular Mn concentration was significantly less than the wild type strain (1/5 for the GB-1-007 strain and 1/59 for the ∆2665 ∆2447 strain). Iron, copper, nickel, and zinc were largely unchanged in the mutant strains when grown in the presence or absence of Mn. 

**Table 1:** Intracellular metal concentration.

**Strain**	**Growth Condition**	**Mn (nmol/mg protein)**	**Fe (nmol/mg protein)**	**Cu (nmol/mg protein)**	**Ni (nmol/mg protein)**	**Zn (nmol/mg protein)**
*P. putida* GB-1	+ Mn	27.6 (±11.6)	43.1 (±5.56)	0.427 (±0.054)	0.054 (±0.008)	2.02 (±0.154)
*P. putida* GB-1	- Mn	0.010 (±0.001)	32.7 (±3.95)	0.164 (±0.009)	0.015 (±001)	1.28 (±0.113)
*P. putida* ∆2665 ∆2447	+ Mn	0.466 (±0.041)	16.4 (±1.11)	0.178 (±0.003)	BD	1.20 (±0.018)
*P. putida* ∆2665 ∆2447	- Mn	BD	21.5 (±1.48)	0.215 (±0.004)	BD	1.31 (±0.025)
*P. putida* GB-1-007	+ Mn	5.19 (±2.77)	17.7 (±3.03)	0.210 (±0.022)	0.022 (±0.005)	1.81 (±0.381)
*P. putida* GB-1-007	- Mn	0.052 (±0.031)	22.8 (±3.72)	0.169 (±0.012)	0.017 (±0.006)	1.43 (±0.108)

Standard deviation of three biological replicates in parentheses. BD = below detection.

The increase in Fe levels of wild type cells grown in Mn added conditions may be from Mn interfering with Fe requiring metabolism causing the cell to perceive Fe starvation. This is in line with other studies where high levels of Mn derepress Fe acquisition genes and repress Fe storage protein genes [29,39]. These findings suggest that when Mn levels are high, cells either need additional Fe or interpret high Mn as a low Fe condition. Alternatively, if Mn transporters are upregulated in response to Mn oxidation, transport of other divalent metals may also occur as many divalent transporters can have broad substrate specificity [40–42].

The increase in intracellular Mn also led to an increase in the intracellular Mn/Fe ratio (Table 2). This ratio may serve as an indicator of how well a cell may survive oxidative stress as Mn can potentially protect cells against oxidative stress and Fe can increase oxidative stress. Although intracellular Fe also increased in the presence of added Mn, it was to a much lesser extent than the increase in intracellular Mn. The increased Mn/Fe ratio was not as high as that found in *Deinococcus radiodurans* [25] (Table 2) which is known to have high intracellular Mn and low Fe, but the increase was significant. 

**Table 2:** Intracellular Mn/Fe Ratios.

**Strain (Growth media)**	**Mn/Fe**	**Reference**
*P. putida* GB-1 (Lept + Mn)	0.64	This work
*P. putida* GB-1 (Lept – Mn)	0.0003	This work
*P. putida* ∆2665 ∆2447 (Lept + Mn)	0.028	This work
*P. putida* ∆2665 ∆2447 (Lept – Mn)	BD	This work
*P. putida* GB1-007 (Lept + Mn)	0.29	This work
*P. putida* GB1-007 (Lept – Mn)	0.002	This work
*P. putida* KT2440 (complex media)	<0.0001	[25]
*Deinococcus radiodurans* (2.5 µM Mn in minimal media)	2.5	[25]
*Deinococcus radiodurans* (complex media)	0.24	[25]
*Deinococcus geothermalis* (complex media)	0.46	[25]

BD = Below Detection

Although the data illustrated that Mn oxidation can increase intracellular Mn, the mechanism is unknown. Mn oxidation may decrease efflux of Mn from the cell or it could be directly linked to uptake. Mn uptake transporters include NRAMP type MntH transporters, SitABC ABC-type transporters, and MntX transporters [43–45]. Although Mn transporters have not been experimentally identified in *P. putida* GB-1, there are candidate ABC type divalent metal transporters including PputGB1_2113-2115 and PputGB1_0132-0135. Coupling oxidation with transport has been demonstrated for Fe transport in yeast whereby the oxidation of Fe(II) to Fe(III) occurs using the multicopper oxidase Fet3p and ferric uptake transporter, Ftr1p [46,47]. Another possibility is that Mn uptake by the cell may occur as Mn(III). Mn oxides have been shown to accumulate extracellularly in *P. putida* GB-1 [17], thus Mn(III) uptake may compete with oxidation to Mn(IV) and could serve as a mechanism to decrease precipitation on the cell surface. 

### Fate of intracellular Mn

Because the significant increase in intracellular Mn may affect metallation of proteins, the association of Mn with cellular protein was determined. Mn has been shown to bind apoferritin [48], and proteins such as bacterioferritin may also bind Mn. The *P. putida* GB-1 genome does contain putative bacterioferritin encoding genes. High levels of Mn may also cause a conversion from Fe based metabolism to Mn based metabolism leading to additional Mn being associated with cellular protein, as such substitutions of Mn for Fe can protect enzymes with solvent exposed Fe cofactors from oxidative stress [49]. To address this, soluble cell free extract from Mn oxidizing cultures was separated into a greater than 3 kDa and a less than 3 kDa fraction. Proteins were retained in the greater than 3 kDa fraction. No protein was detected in the less than 3 kDa fraction. Approximately 10% of the Mn was present with the protein (Table 3). The majority of the Mn was present in the small molecule fraction, suggesting it was not associated with protein although it could be associated with small peptides or may have been released by especially labile proteins during sample preparation. In contrast, a significant amount of the iron was associated with the protein fraction. This suggests that the increased intracellular Mn is free aqueous Mn^2+^ (Mn(H_2_O)_6_^2+^) or complexed with small molecules such as phosphates, small peptides, nucleotides, or organic acids. Nucleotides are one of the most abundant metabolites detected in a metabolite screen of closely related *P. putida* S12 [50]. In addition, organic acids were also detected [50], suggesting the *P. putida* GB-1 is likely to have small molecules available to complex Mn. The Mn levels determined for *P. putida* GB-1 were lower in cell extract compared to whole cells, and this is likely due to the oxidation and precipitation of Mn resulting from in vitro Mn oxidation [17] once the cells are broken. 

**Table 3:** Intracellular Mn and Fe in total cell-free extracts, < 3K cell extract fraction, and > 3K cell extract fraction.

	**Mn**	**Fe**
Soluble cell-free extract	4.95 ± 1.25 µM	19.3 ± 1.78 µM
> 3K cell extract fraction	0.54 ±0.15 µM	7.62 ±0.96 µM
< 3K cell extract fraction	4.11 ±1.13 µM	6.13 ± 1.58 µM

± standard deviations from 3 biological replicates

### Mn(II) oxidation protects cells

Mn(II) and Mn oxides have been shown to react with and scavenge both hydrogen peroxide and superoxide [51–54]. Several studies have identified an increase in protection against reactive oxygen species when cells have high intracellular Mn [55]. Two well-known examples would be *Deinococcus radiodurans* which uses Mn in combination with phosphate and peptides to protect protein from oxidative stress [25,56] and *Lactobacillus plantarum* which can functionally substitute high intracellular manganese for superoxide dismutase activity [57]. Therefore, the extent of oxidative stress under Mn oxidizing and non-oxidizing conditions was determined by examining cell survival and the level of protein carbonylation. Only hydrogen peroxide was used for oxidative stress survival studies as the superoxide generator paraquat can be metabolized by a *P. putida* strain [58], may not induce genes involved in oxidative protection [59], and did not kill the cells (data not shown). 

**Figure 1:**
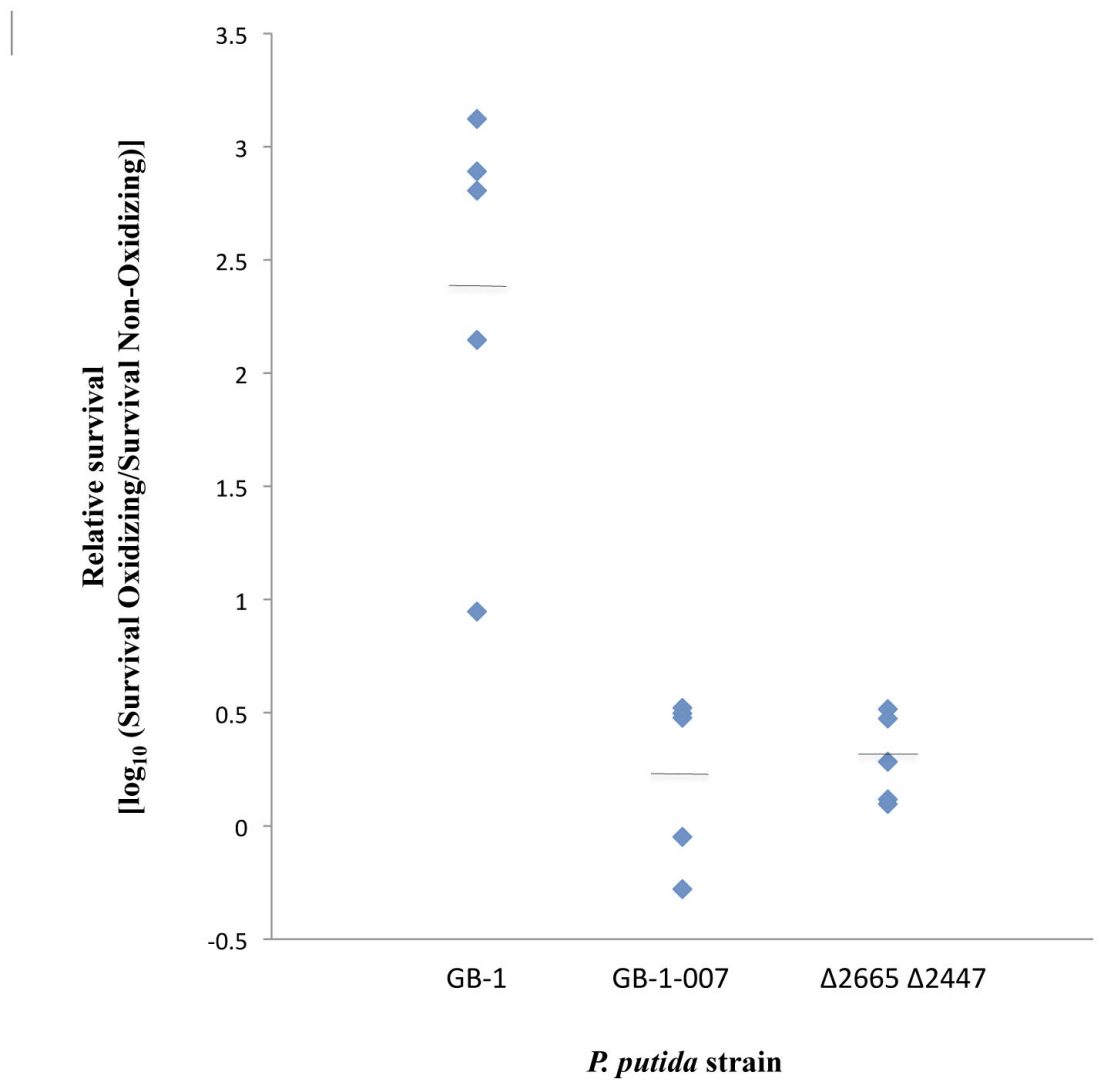
Survival of *P. putida* following hydrogen peroxide stress. *P. putida* GB-1, GB-1-007, and ∆2665 ∆2447 strains were exposed to 16.1 mM hydrogen peroxide for 30 minutes. Relative survival was calculated as the log_10_ of the percent survival rate of the oxidizing (+ Mn) culture divided by the percent survival rate of the non-oxidizing (-Mn) culture. The horizontal lines represent the mean value of the data sets. The mean relative survival of *P*. *putida* GB-1 is significantly higher than the mean relative survival of *P*. *putida* GB-1-007 and *P*. *putida* ∆2665 ∆2447 (P < 0.05, n=5) as determined by the Student’s t-test. Although the relative survival varied by experiment, *P. putida* GB1 consistently showed greater relative survival than the non-oxidizing mutants.

*P. putida* GB-1 was grown under Mn oxidizing (+100 µM Mn) and non-oxidizing conditions (no Mn addition) and then stressed with hydrogen peroxide. MSTP media was used to avoid the cell clumping common with the complex Lept media that led to poor reproducibility of CFU counts. *P. putida* GB-1 grown in the presence of Mn, produced Mn oxides, had higher intracellular Mn concentrations, and had survival levels greater than the Mn oxidizing mutants *P. putida* GB-1-007 and *P. putida* ∆2665 ∆2447 (Figure 1) when grown in the same Mn(II) containing media. The increase in survival correlates with the presence of Mn oxides and is not related to Mn(II) as the same extent of increased survival was not found with *P. putida* GB-1-007 and *P. putida* ∆2665 ∆2447. The increase in survival in the presence of Mn oxides may be related to the ability of Mn oxides to react with hydrogen peroxide [51] as Mn oxides were reduced upon the addition of hydrogen peroxide (data not shown). 

**Figure 2:**
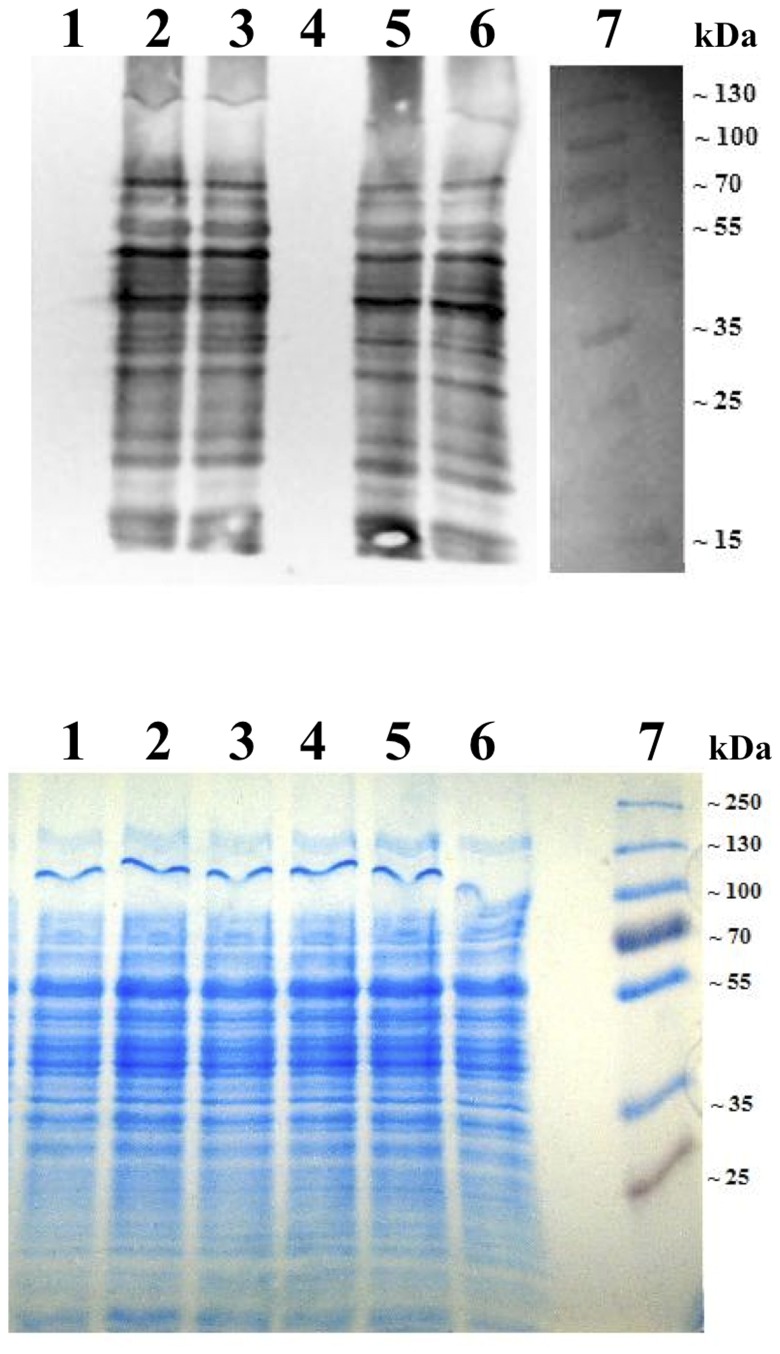
Protein carbonylation following oxidative stress. A) Oxyblot and B) Coomassie stained SDS-PAGE of hydrogen peroxide treated *P. putida* GB-1 grown under oxidizing (+Mn) and non-oxidizing (-Mn) conditions. Stationary phase cells were treated with 0.6 mM hydrogen peroxide for 30 minutes. The extent of protein carbonylation in cells oxidatively stressed in the presence or absence of Mn oxides was equivalent. Lane 1: negative control for non-oxidizing conditions; Lane 2,3: non-oxidizing conditions; Lane 4: Mn oxidizing negative control; Lane 5,6: Mn oxidizing conditions; Lane 7: Protein standard. The negative control was not derivatized with dinitrophenylhydrazine.

Protein carbonylation, an indicator of cellular oxidative damage, was determined for cells grown under Mn oxidizing (+Mn) and non oxidizing (-Mn) conditions. Protein was analyzed from stationary phase *P. putida* GB-1 in the absence of oxidative stress and following exposure to hydrogen peroxide and paraquat. In the absence of oxidative stress, protein from stationary phase cells grown under Mn oxidizing conditions had a slight decrease in protein carbonylation compared to non-oxidizing cultures (data not shown). But, there was no discernable difference in the amount of protein oxidation between Mn oxidizing (+Mn) and non-oxidizing (-Mn) conditions when cells were oxidatively stressed with hydrogen peroxide (0.6 mM) (Figure 2) or paraquat (5 mM). Higher concentrations of hydrogen peroxide (6mM, 60 mM) did not increase the extent of protein carbonylation. 

The lack of protein protection against ROS in Mn oxidizing cultures is somewhat unexpected, but there are several reasons why this may occur. The intracellular concentration of Mn may not be as important as the presence of specific ligands to complex Mn inside the cell [53,55–57,60–66]. The increase in intracellular Mn in Mn oxidizing *P. putida* GB-1 is also accompanied by an increase in intracellular Fe that can produce additional oxidative damage through Fenton chemistry. In addition, oxidative stress resistance or other regulatory and signaling pathways may be up or down regulated during Mn oxidation and increased intracellular Mn [65,67]. Thus, the survival benefit may not be related to intracellular Mn concentrations, but to the external reaction of Mn oxides with hydrogen peroxide. 

In summary, we have found an intriguing link between Mn oxidation and intracellular Mn levels. Our data suggests that Mn oxidation can affect Mn homeostasis and enhances the survival of cells experiencing hydrogen peroxide stress.

## References

[1] Tebo BM, Johnson HA, McCarthy JK, Templeton AS (2005) Geomicrobiology of manganese(II) oxidation. Trends Microbiol 13: 421-428. doi:10.1016/j.tim.2005.07.009. PubMed: 16054815. 16054815

[2] Tebo BM, Bargar JR, Clement BG, Dick GJ, Murray KJ et al. (2004) Biogenic manganese oxides: properties and mechanisms of formation. Annu Rev Earth Planet Sci 32: 287-328. doi:10.1146/annurev.earth.32.101802.120213.

[3] Santelli CM, Pfister DH, Lazarus D, Sun L, Burgos WD et al. (2010) Promotion of Mn(II) oxidation and remediation of coal mine drainage in passive treatment systems by diverse fungal and bacterial communities. Appl Environ Microbiol 76: 4871-4875. doi:10.1128/AEM.03029-09. PubMed: 20495049. 20495049 PMC2901711

[4] Charmichael MJ, Carmichael SK, Santelli CM, Strom A, Brauer SL (2013) Mn(II)-oxidizing bacteria are abundant and environmentally relevant members of the ferromanganese deposits in caves of the upper Tennessee River Basin. Geomicrobiol J 30: 779-800. doi:10.1080/01490451.2013.769651.

[5] Nealson KH, Tebo BM, Rosson RA (1988) Occurrence and Mechanisms of Microbial Oxidation of Manganese. Adv Appl Microbiol 33: 279-318. doi:10.1016/S0065-2164(08)70209-0.

[6] Laha S, Luthy RG (1990) Oxidation of Aniline and Other Primary Aromatic-Amines by Manganese-Dioxide. Environ Sci Technol 24: 363-373. doi:10.1021/es00073a012.

[7] Miyata N, Tani Y, Sakata M, Iwahori K (2007) Microbial manganese oxide formation and interaction with toxic metal ions. J Biosci Bioeng 104: 1-8. doi:10.1263/jbb.104.1. PubMed: 17697976. 17697976

[8] Spiro TG, Bargar JR, Sposito G, Tebo BM (2010) Bacteriogenic manganese oxides. Acc Chem Res 43: 2-9. doi:10.1021/ar800232a. PubMed: 19778036. 19778036

[9] Geszvain K, Butterfield C, Davis RE, Madison AS, Lee SW et al. (2012) The molecular biogeochemistry of manganese(II) oxidation. Biochem Soc Trans 40: 1244-1248. doi:10.1042/BST20120229. PubMed: 23176462. 23176462

[10] Geszvain K, Yamaguchi A, Maybee J, Tebo BM (2011) Mn(II) oxidation in *Pseudomonas* *putida* GB-1 is influenced by flagella synthesis and surface substrate. Arch Microbiol 193: 605-614. doi:10.1007/s00203-011-0702-0. PubMed: 21479918. 21479918

[11] De Vrind J, De Groot A, Brouwers GJ, Tommassen J, De Vrind-De Jong E (2003) Identification of a novel Gsp-related pathway required for secretion of the manganese-oxidizing factor of *Pseudomonas* *putida* strain GB-1. Mol Microbiol 47: 993-1006. doi:10.1046/j.1365-2958.2003.03339.x. PubMed: 12581354. 12581354

[12] Brouwers G, de Vrind JPM, Corstjens PLAM, de Vrind-de Jong EW (1998) Involvement of genes of the two-step protein secretion pathway in the transport of the manganese-oxidizing factor across the outer membrane of *Pseudomonas* *putida* strain GB-1. Am Mineral 83: 1573-1582.

[13] Geszvain K, Tebo BM (2010) Identification of a two-component regulatory pathway essential for Mn(II) oxidation in *Pseudomonas* *putida* GB-1. Appl Environ Microbiol 76: 1224-1231. doi:10.1128/AEM.02473-09. PubMed: 20038702. 20038702 PMC2820985

[14] de Vrind JPM, Brouwers GJ, Corstjens PLAM, den Dulk J, de Vrind-de Jong EW (1998) The cytochrome c maturation operon is involved in manganese oxidation in *Pseudomonas* *putida* GB-1. Appl Environ Microbiol 64: 3556-35562. PubMed: 9758767. 9758767 10.1128/aem.64.10.3556-3562.1998 PMC106464

[15] Caspi R, Tebo BM, Haygood MG (1998) c-type cytochromes and manganese oxidation in *Pseudomonas* *putida* MnB1. Appl Environ Microbiol 64: 3549-3555. PubMed: 9758766. 9758766 10.1128/aem.64.10.3549-3555.1998 PMC106463

[16] Geszvain K, McCarthy JK, Tebo BM (2013) Elimination of manganese(II,III) oxidation in *Pseudomonas* *putida* GB-1 by a double knockout of two putative multicopper oxidase genes. Appl Environ Microbiol 79: 357-366. doi:10.1128/AEM.01850-12. PubMed: 23124227. 23124227 PMC3536112

[17] Okazaki M, Sugita T, Shimizu M, Ohode Y, Iwamoto K et al. (1997) Partial purification and characterization of manganese-oxidizing factors of *Pseudomonas* *fluorescens* GB-1. Appl Environ Microbiol 63: 4793-4799. PubMed: 9406397. 9406397 10.1128/aem.63.12.4793-4799.1997 PMC168802

[18] Johnson HA, Tebo BM (2008) In vitro studies indicate a quinone is involved in bacterial Mn(II) oxidation. Arch Microbiol 189: 59-69. PubMed: 17673976. 17673976 10.1007/s00203-007-0293-y PMC2721854

[19] Tebo BM, Ghiorse WC, vanWaasbergen LG, Siering PL, Caspi R (1997) Bacterially mediated mineral formation: Insights into manganese(II) oxidation from molecular genetic and biochemical studies. Geomicrobiol Interact Between Microbes Miner 35: 225-266.

[20] Sunda WG, Kieber DJ (1994) Oxidation of humic substances by manganese oxides yields low molecular weight organic substrates. Nature 367: 62-64. doi:10.1038/367062a0.

[21] Ehrlich HL, Salerno JC (1990) Energy coupling in Mn-2+ oxidation by a marine bacterium. Arch Microbiol 154: 12-17.

[22] Brouwers GJ, Vijgenboom E, Corstjens PLAM, De Vrind JPM, de Vrind-de Jong EW (2000) Bacterial Mn2+ oxidizing systems and multicopper oxidases: An overview of mechanisms and functions. Geomicrobiol J 17: 1-24. doi:10.1080/014904500270459.

[23] Ghiorse WC (1984) Biology of iron- and manganese-depositing bacteria. Annu Rev Microbiol 38: 515-550. doi:10.1146/annurev.micro.38.1.515. PubMed: 6388499. 6388499

[24] Archibald FS, Fridovich I (1981) Manganese and Defenses against Oxygen-Toxicity in *Lactobacillus* *plantarum* . J Bacteriol 145: 442-451. PubMed: 6257639. 6257639 10.1128/jb.145.1.442-451.1981 PMC217292

[25] Daly MJ, Gaidamakova EK, Matrosova VY, Vasilenko A, Zhai M et al. (2004) Accumulation of Mn(II) in *Deinococcus* *radiodurans* facilitates gamma-radiation resistance. Science 306: 1025-1028. doi:10.1126/science.1103185. PubMed: 15459345. 15459345

[26] Li C, Tao J, Mao D, He C (2011) A novel manganese efflux system, YebN, is required for virulence by *Xanthomonas* *oryzae* *pv.* *oryzae* . PLOS ONE 6: e21983. doi:10.1371/journal.pone.0021983. PubMed: 21789199. 21789199 PMC3136493

[27] Hohle TH, O'Brian MR (2012) Manganese is required for oxidative metabolism in unstressed *Bradyrhizobium* *japonicum* cells. Mol Microbiol 84: 766-777. doi:10.1111/j.1365-2958.2012.08057.x. PubMed: 22463793. 22463793 PMC3345040

[28] Veyrier FJ, Boneca IG, Cellier MF, Taha MK (2011) A novel metal transporter mediating manganese export (MntX) regulates the Mn to Fe intracellular ratio and *Neisseria* *meningitidis* virulence. PLOS Pathog 7: e1002261. 21980287 10.1371/journal.ppat.1002261 PMC3182930

[29] Waters LS, Sandoval M, Storz G (2011) The *Escherichia* *coli* MntR miniregulon includes genes encoding a small protein and an efflux pump required for manganese homeostasis. J Bacteriol 193: 5887-5897. doi:10.1128/JB.05872-11. PubMed: 21908668. 21908668 PMC3194919

[30] Jakubovics NS, Valentine RA (2009) A new direction for manganese homeostasis in bacteria: identification of a novel efflux system in *Streptococcus* *pneumoniae* . Mol Microbiol 72: 1-4. doi:10.1111/j.1365-2958.2009.06637.x. PubMed: 19226325. 19226325

[31] Champion OL, Karlyshev A, Cooper IA, Ford DC, Wren BW et al. (2010) *Yersinia* *pseudotuberculosis* mntH functions in intracellular manganese accumulation, which is essential for virulence and survival in cells expressing functional Nramp1. Microbiology 157: 1115-1122. PubMed: 21183572. 21183572 10.1099/mic.0.045807-0

[32] Sun H, Xu G, Zhan H, Chen H, Sun Z et al. (2010) Identification and evaluation of the role of the manganese efflux protein in *Deinococcus* *radiodurans* . BMC Microbiol 10: 319. doi:10.1186/1471-2180-10-319. PubMed: 21156049. 21156049 PMC3016326

[33] Hohle TH, O'Brian MR (2009) The mntH gene encodes the major Mn(2+) transporter in *Bradyrhizobium* *japonicum* and is regulated by manganese via the Fur protein. Mol Microbiol 72: 399-409. doi:10.1111/j.1365-2958.2009.06650.x. PubMed: 19298371. 19298371 PMC2675660

[34] Brouwers GJ, de Vrind JPM, Corstjens PLAM, Cornelis P, Baysse C et al. (1999) *cumA*, a gene encoding a multicopper oxidase, is involved in Mn^2+^ oxidation in *Pseudomonas* *putida* GB-1. Appl Environ Microbiol 65: 1762-1768. PubMed: 10103278. 10103278 10.1128/aem.65.4.1762-1768.1999 PMC91248

[35] Boogerd FC, de Vrind JP (1987) Manganese oxidation by *Leptothrix* *discophora* . J Bacteriol 169: 489-494. PubMed: 3804969. 3804969 10.1128/jb.169.2.489-494.1987 PMC211803

[36] Klock HE, Koesema EJ, Knuth MW, Lesley SA (2007) Combining the polymerase incomplete primer extension method for cloning and mutagenesis with microscreening to accelerate structural genomics efforts. Proteins 71: 982-994. PubMed: 18004753. 10.1002/prot.21786 18004753

[37] Parikh SJ, Chorover J (2005) FTIR spectroscopic study of biogenic Mn-oxide formation by *Pseudomonas* *putida* GB-1. Geomicrobiol J 22: 207-218. doi:10.1080/01490450590947724.

[38] Tebo BM, Clement BG, Dick GJ (2007) Biotransformations of Manganese. In: Hurst CJ Manual of Environmental Microbiology. 3 ed. Washington, D.C.: ASM Press pp 1223-1238.

[39] Yamamoto K, Ishihama A, Busby SJ, Grainger DC (2011) The *Escherichia* *coli* K-12 MntR miniregulon includes dps, which encodes the major stationary-phase DNA-binding protein. J Bacteriol 193: 1477-1480. doi:10.1128/JB.01230-10. PubMed: 21239586. 21239586 PMC3067631

[40] Illing AC, Shawki A, Cunningham CL, Mackenzie B (2012) Substrate profile and metal-ion selectivity of human divalent metal-ion transporter-1. J Biol Chem 287: 30485-30496. doi:10.1074/jbc.M112.364208. PubMed: 22736759. 22736759 PMC3436370

[41] Grass G, Franke S, Taudte N, Nies DH, Kucharski LM et al. (2005) The metal permease ZupT from *Escherichia* *coli* is a transporter with a broad substrate spectrum. J Bacteriol 187: 1604-1611. doi:10.1128/JB.187.5.1604-1611.2005. PubMed: 15716430. 15716430 PMC1064025

[42] Makui H, Roig E, Cole ST, Helmann JD, Gros P et al. (2000) Identification of the *Escherichia* *coli* K-12 Nramp orthologue (MntH) as a selective divalent metal ion transporter. Mol Microbiol 35: 1065-1078. doi:10.1046/j.1365-2958.2000.01774.x. PubMed: 10712688. 10712688

[43] Cellier MF (2012) Nramp: from sequence to structure and mechanism of divalent metal import. Curr Top Membr 69: 249-293. doi:10.1016/B978-0-12-394390-3.00010-0. PubMed: 23046654. 23046654

[44] Klein JS, Lewinson O (2011) Bacterial ATP-driven transporters of transition metals: physiological roles, mechanisms of action, and roles in bacterial virulence. Metallomics 3: 1098-1108. doi:10.1039/c1mt00073j. PubMed: 21901186. 21901186

[45] Green RT, Todd JD, Johnston AW (2013) Manganese uptake in marine bacteria; the novel MntX transporter is widespread in *Roseobacters,* *Vibrios,* *Alteromonadales* and the SAR11 and SAR116 clades. ISME J 7: 581-591. doi:10.1038/ismej.2012.140. PubMed: 23190726. 23190726 PMC3578558

[46] Stearman R, Yuan DS, Yamaguchi-Iwai Y, Klausner RD, Dancis A (1996) A permease-oxidase complex involved in high-affinity iron uptake in yeast. Science 271: 1552-1557. doi:10.1126/science.271.5255.1552. PubMed: 8599111. 8599111

[47] Kwok EY, Severance S, Kosman DJ (2006) Evidence for iron channeling in the Fet3p-Ftr1p high-affinity iron uptake complex in the yeast plasma membrane. Biochemistry 45: 6317-6327. doi:10.1021/bi052173c. PubMed: 16700543. 16700543

[48] Macara IG, Hoy TG, Harrison PM (1973) The formation of ferritin from apoferritin. Inhibition and metal ion-binding studies. Biochem J 135: 785-789. PubMed: 4798313. 4798313 10.1042/bj1350785 PMC1165895

[49] Sobota JM, Imlay JA (2011) Iron enzyme ribulose-5-phosphate 3-epimerase in *Escherichia* *coli* is rapidly damaged by hydrogen peroxide but can be protected by manganese. Proc Natl Acad Sci U S A 108: 5402-5407. doi:10.1073/pnas.1100410108. PubMed: 21402925. 21402925 PMC3069151

[50] van der Werf MJ, Overkamp KM, Muilwijk B, Koek MM, van der Werff-van der Vat BJ et al. (2008) Comprehensive analysis of the metabolome of *Pseudomonas* *putida* S12 grown on different carbon sources. Mol Biosyst 4: 315-327. doi:10.1039/b717340g. PubMed: 18354785. 18354785

[51] Do SH, Batchelor B, Lee HK, Kong SH (2009) Hydrogen peroxide decomposition on manganese oxide (pyrolusite): kinetics, intermediates, and mechanism. Chemosphere 75: 8-12. doi:10.1016/j.chemosphere.2008.11.075. PubMed: 19136141. 19136141

[52] Stadtman ER, Berlett BS, Chock PB (1990) Manganese-dependent disproportionation of hydrogen peroxide in bicarbonate buffer. Proc Natl Acad Sci U S A 87: 384-388. doi:10.1073/pnas.87.1.384. PubMed: 2296593. 2296593 PMC53268

[53] Berlett BS, Chock PB, Yim MB, Stadtman ER (1990) Manganese(II) catalyzes the bicarbonate-dependent oxidation of amino acids by hydrogen peroxide and the amino acid-facilitated dismutation of hydrogen peroxide. Proc Natl Acad Sci U S A 87: 389-393. doi:10.1073/pnas.87.1.389. PubMed: 2296594. 2296594 PMC53269

[54] Learman DR, Voelker BM, Vazquez-Rodriguez AI, Hansel CM (2011) Formation of manganese oxides by bacterially generated superoxide. Nat Geosci 4: 95-98. doi:10.1038/ngeo1055.

[55] Aguirre JD, Culotta VC (2012) Battles with iron: manganese in oxidative stress protection. J Biol Chem 287: 13541-13548. doi:10.1074/jbc.R111.312181. PubMed: 22247543. 22247543 PMC3340200

[56] Daly MJ, Gaidamakova EK, Matrosova VY, Kiang JG, Fukumoto R et al. (2010) Small-molecule antioxidant proteome-shields in *Deinococcus* *radiodurans* . PLOS ONE 5: e12570. doi:10.1371/journal.pone.0012570. PubMed: 20838443. 20838443 PMC2933237

[57] Archibald FS, Fridovich I (1982) The scavenging of superoxide radical by manganous complexes: in vitro. Arch Biochem Biophys 214: 452-463. doi:10.1016/0003-9861(82)90049-2. PubMed: 6284026. 6284026

[58] Kopytko M, Chalela G, Zauscher F (2002) Biodegradation of two commercial herbicides (Gramoxone and Matancha) by the bacteria *Pseudomonas* *putida* . Electron J Biotechnol 5: 182-195.

[59] Carr RJ, Bilton RF, Atkinson T (1986) Toxicity of paraquat to microorganisms. Appl Environ Microbiol 52: 1112-1116. PubMed: 3098166. 3098166 10.1128/aem.52.5.1112-1116.1986 PMC239182

[60] Yim MB, Berlett BS, Chock PB, Stadtman ER (1990) Manganese(II)-bicarbonate-mediated catalytic activity for hydrogen peroxide dismutation and amino acid oxidation: detection of free radical intermediates. Proc Natl Acad Sci U S A 87: 394-398. doi:10.1073/pnas.87.1.394. PubMed: 2153299. 2153299 PMC53270

[61] Barnese K, Gralla EB, Valentine JS, Cabelli DE (2012) Biologically relevant mechanism for catalytic superoxide removal by simple manganese compounds. Proc Natl Acad Sci U S A 109: 6892-6897. doi:10.1073/pnas.1203051109. PubMed: 22505740. 22505740 PMC3344976

[62] McNaughton RL, Reddi AR, Clement MH, Sharma A, Barnese K et al. (2010) Probing in vivo Mn2+ speciation and oxidative stress resistance in yeast cells with electron-nuclear double resonance spectroscopy. Proc Natl Acad Sci U S A 107: 15335-15339. doi:10.1073/pnas.1009648107. PubMed: 20702768. 20702768 PMC2932569

[63] Barnese K, Gralla EB, Cabelli DE, Valentine JS (2008) Manganous phosphate acts as a superoxide dismutase. J Am Chem Soc 130: 4604-4606. doi:10.1021/ja710162n. PubMed: 18341341. 18341341

[64] Granger AC, Gaidamakova EK, Matrosova VY, Daly MJ, Setlow P (2011) Effects of Mn and Fe levels on *Bacillus* *subtilis* spore resistance and effects of Mn2+, other divalent cations, orthophosphate, and dipicolinic acid on protein resistance to ionizing radiation. Appl Environ Microbiol 77: 32-40. doi:10.1128/AEM.01965-10. PubMed: 21057011. 21057011 PMC3019732

[65] Anjem A, Varghese S, Imlay JA (2009) Manganese import is a key element of the OxyR response to hydrogen peroxide in *Escherichia* *coli* . Mol Microbiol 72: 844-858. doi:10.1111/j.1365-2958.2009.06699.x. PubMed: 19400769. 19400769 PMC2776087

[66] Culotta VC, Daly MJ (2013) Manganese Complexes: Diverse Metabolic Routes to Oxidative Stress Resistance in Prokaryotes and Yeast. Antioxid Redox Signal 19: 933-944. doi:10.1089/ars.2012.5093. PubMed: 23249283. 23249283 PMC3763226

[67] Galiazzo F, Pedersen JZ, Civitareale P, Schiesser A, Rotilio G (1989) Manganese accumulation in yeast cells. Electron-spin-resonance characterization and superoxide dismutase activity. Biol Met 2: 6-10. doi:10.1007/BF01116194. PubMed: 2562042. 2562042

